# Perception and Practice of Workplace Violence Prevention and Its Associated Factors among Employers at Healthcare Facilities in Melaka, Malaysia

**DOI:** 10.3390/ijerph20042900

**Published:** 2023-02-07

**Authors:** Mohd Nizam Mohamad Yazid, Nik Rosmawati Nik Husain, Aziah Daud, Yelmizaitun Osman, Normazura Mustapa, Azlihanis Abdul Hadi

**Affiliations:** 1 Department of Community Medicine, School of Medical Sciences, Universiti Sains Malaysia, Kubang Kerian 16150, Kelantan, Malaysia; 2 Kelantan State Health Department, Kota Bharu 15590, Kelantan, Malaysia; 3 Melaka State Health Department, Melaka International Trade Centre, Malacca 75450, Melaka, Malaysia; 4 Ministry of Health Malaysia, Federal Government Administrative Centre, Putrajaya 62590, Selangor, Malaysia

**Keywords:** workplace violence prevention, perception, practice, healthcare employers, associated factors

## Abstract

Workplace violence (WPV) is a major public health concern, especially among healthcare workers. There is a negative perception and poor practice of healthcare employers towards WPV prevention. This study aims to determine the perception and practice towards WPV prevention and its associated factors among healthcare employers in Melaka, Malaysia. A cross-sectional study was conducted by recruiting 162 healthcare employers, using a validated questionnaire and utilised linear regression analysis. The participants had a mean percentage of 67.2% for perception and 80% for practice towards WPV prevention. The perception towards WPV prevention is associated with the following characteristics: female (adjusted ß = −3.95; 95% CI: −7.81, −0.09; *p* = 0.045), Indian ethnicity (adjusted ß = 16.04; 95% CI: 2.34, 29.74; *p* = 0.022), other ethnicities (adjusted ß = 25.71; 95% CI: 8.94, 42.47; *p* = 0.003), degree holder (adjusted ß = 4.35; 95% CI: 0.15, 8.54; *p* = 0.042), masters holder (adjusted ß = 7.63; 95% CI: 1.11, 14.14; *p* = 0.022), and enough funds (adjusted ß = −5.46; 95% CI: −9.25, −1.67; *p* = 0.005). Meanwhile, the practice towards WPV prevention is significantly associated with Chinese ethnicity (adjusted ß = −9.25; 95% CI: −18.36, −0.14; *p* = 0.047), Indian ethnicity (adjusted ß = −14.97; 95% CI: −29.48, −0.46; *p* = 0.043), other ethnicities (adjusted ß = 23.55; 95% CI: 5.59, 41.51; *p* = 0.011), degree holder (adjusted ß = −4.41; 95% CI: −8.67, −0.14; *p* = 0.043), and availability of standard operating procedure for reporting WPV (adjusted ß = 6.07; 95% CI: 1.58, 10.57; *p* = 0.008). The high perception and practice towards WPV prevention and its associated factors among healthcare employers provide evidence-based input to improve the existing measures for WPV prevention.

## 1. Introduction

Workplace violence (WPV), as adapted from the European Commission Directorates-General V (EU DG-V), is defined as the incident when workers are abused, threatened, or attacked in conditions connected with their work, including their commute to and from work, posing a threat to their health, safety or well-being, either explicitly or implicitly [1]. The recognised forms include physical injuries, verbal abuse, racial abuse, bullying, and sexual harassment [2]. Meanwhile, WPV can be classified into four types according to different perpetrators: Type I (Criminal intent), Type II (Patient/Visitor), Type III (Worker-on-worker), and Type IV (Organisational) [3].

Globally, workplace violence in healthcare facilities is reportedly high in prevalence and keeps increasing [4,5,6,7,8]. Similarly, WPV against healthcare workers (HCWs) is on the rise in Malaysia. The reported WPV in a public hospital was 38% [9] and in a primary care and community-based setting was 24.3% [10]. It has been found that 70% of HCWs in Malaysia have experienced verbal abuse, 33% physical abuse, 25% bullying, and 4% sexual harassment in the workplace [11]. According to the Ministry of Health Malaysia, HCWs reportedly are most prone to Type II (Patient/Visitor) WPV in healthcare facilities [11].

WPV would lower the standard of medical care by affecting doctors’ attitudes toward their work and deter them from providing their patients with the best care possible [12]. Previous studies reported that medical professionals avoided high-risk procedures to avoid the fury that would arise in the event of a negative outcome [13]. WPV against HCWs negatively impacts their physical health, such as cardiovascular disease [14,15] and mental health, including tiredness and reduced concentration during work time [16]. Hence, these studies emphasised that such WPV events could impair both health service quality and the health of HCWs, further jeopardising community health.

Locally, there is legislation on WPV developed by organisations, such as general guidelines for the prevention of WPV by the Department of Occupational Safety and Health Malaysia [2]. In addition, the Ministry of Health Malaysia has also launched guidelines and training modules on WPV prevention specifically for HCWs [17,18]. However, it is unfortunate that the prevalence of WPV against HCWs in Malaysia continues to increase, even though guidelines and training modules have been launched and implemented in healthcare facilities to prevent WPV.

Progressive measures should be conducted immediately to overcome the issue of WPV in healthcare facilities. Factors associated with WPV have been studied numerously, but factors for WPV prevention still have limited published data in Malaysia or throughout the world. Nevertheless, a book by Ferris and Murphy [19] highlights seven components of a comprehensive WPV prevention plan: the hiring process, communication, policies, reporting processes, training, physical security, and risk management team. However, these components were explicitly written for the practitioner within an organisation, not tailored specifically to a health institution. A scoping review done by Morphet et al. [20] has summarised that interventions, which reduced WPV included increasing visibility, aggression management teams, consumer risk assessment, and staff education. Post-incident support increased the incidence of WPV, whereas no evidence was found concerning incident reporting or zero-tolerance policies. Therefore, exploring these variables concerning WPV prevention in healthcare facilities is crucial.

Employers’ psychometrics is another measure that should be addressed in WPV as it is essential for the execution of WPV prevention. This is because although the prevention of WPV is well-known, the interventions are not consistently implemented. For example, a previous study showed that employers’ practice of regularly evaluating their employees’ performance and allowing opportunities to discuss grievances were influential in developing WPV prevention [21]. In contrast, a study also showed that employers did not encourage staff to do formal incident reporting post-violence episodes at the workplace [22]. In addition, Leymann [23] highlighted that organisational factors and leadership problems caused workplace bullying.

Therefore, a new perspective on WPV should be sought, particularly the perception and practice towards WPV prevention among employers in healthcare facilities. This study aimed to assess the perception and practice towards WPV prevention and its associated factors among healthcare employers in Melaka.

## 2. Materials and Methods

### 2.1. The Study Design and Participants Selection

A cross-sectional study was conducted between February and July 2022 in Melaka, Malaysia. The study involved five categories of healthcare workplaces: hospitals, health clinics, dental clinics, district health offices, and district dental offices. Employers in healthcare facilities, who had worked at least twelve months in the current workplace, and representatives from any of these three levels: director of the organisation, location supervisor, and those involved in the Occupational Safety & Health Committee (OSHC) were invited for the study. The sample size estimation was 162 participants. Selection was made from the five different workplace categories and the employers’ stratum. We gathered a list of employers in each workplace category during the initial recruitment process. This involved 3 hospitals, 3 district health offices, 3 district dental offices, 33 health clinics, and 20 dental clinics available for selection. The number of participants required for each stratum of employers from each workplace category was determined using stratified proportionate sampling formula. Then, participant selection was made using simple random sampling. Consenting participants were given an online questionnaire via email and an online messaging platform.

### 2.2. Research Instrument: The Perception and Practice of Workplace Violence Prevention Questionnaire

This research used the Malay version of the Perception and Practice of Workplace Violence Prevention Questionnaire (PPWVP) questionnaire, which comprised 56 items constructed under the domains of perception (33 items) and practice (23 items). The perception domain consists of9 factors: form of WPV (8 items), causes of WPV (3 items), impacts of WPV (2 items), benefits of WPV prevention (5 items), barriers to WPV prevention (5 items), high-strain job characteristics (3 items), reaction to WPV (3 items), WPV protection (2 items), and WPV prevention encouragement (2 items). In contrast, the practice domain consists of 4 factors: workplace safety (3 items), WPV prevention implementation (15 items), WPV reporting (2 items), and managerial role (3 items). All the questions were close-ended and rated on a 5-point Likert scale ranging from 1 (*strongly disagree*) to 5 (*strongly agree*). The higher scores indicate a better perception and practice towards WPV prevention.

The item content validation index (I-CVI) values of content validation for all items in both domains were above 0.78, whereas the item face validation index (I-FVI) values of face validation for both domains were above 0.80. In exploratory factor analysis, all items load above 0.6 in their respective factor, Bartlett’s test of sphericity was significant for both domains (*p* < 0.001), and the Kaiser-Meyer-Olkin Measure was 0.879 for the perception domain and 0.941 for the practice domain, with Cronbach’s Alpha coefficient of reliability test ranged from 0.71 to 0.92 and from 0.82 to 0.97 for the perception and practice domains, respectively. Meanwhile, the fit indices of CFA were χ^2^ = 2092.6 (*p* < 0.001), SRMR = 0.053, RMSEA = 0.042, CFI = 0.928, and TLI = 0.920; the factor loadings for all items were above 0.6 with Raykov’s rho coefficients above 0.70.

The questionnaire was created in an electronic way using Google Forms due to the COVID-19 outbreak and the need to practice strict standard operating procedures to minimise exposure and keep social distance in healthcare facilities. The Google Forms link was sent to the participants through email and WhatsApp applications. Upon clicking on the link, the participants were directed to the survey’s entry page, which contained information on the objectives of the study, terms of participation, data privacy, and a consent form on the first page. An explicit instruction was stated at the beginning of the questionnaire to clear the participant’s doubts. The participants were asked to complete the survey in one session, which took 15 to 20 minutes. The participants were able to access the survey and complete it on a computer or a mobile device. 

Once the Google Forms link was sent to the participants, their responses were traced. A gentle reminder was sent to the participants if no response was received after one week. No response after one month was considered missing. The raw data from Google Forms were exported to IBM Statistical Program for Social Sciences (SPSS) version 26 software for data cleaning and further analysis. The data collected were stored electronically and password protected.

### 2.3. Operational Definition

For the study, we have adopted the following operational definitions relating to employers’ status. The operational definition for director of the organisation is the employer who oversees the healthcare facilities, including the hospital director and medical officer in charge of healthcare facilities. A location supervisor is referred to the employer who oversees the respective department in healthcare facilities, including the head of the department, senior assistant medical officer, environmental health officer, and matron. Meanwhile, OSHC in healthcare facilities is defined as a committee consisting of safety and health officer, workers, and representatives of the organisation to improve health and safety at work. 

### 2.4. Statistical Analysis

Data analysis was performed using SPSS software. A descriptive analysis was done to determine the characteristics of the participants. The mean and standard deviation (SD) were used to describe continuous variables, whereas frequency and percentage (%) were used to describe categorical variables. The mean percentage was calculated to measure the perception score and practice score. It was calculated by first computing the mean of the total score for each perception and practice domain, then dividing the mean by the maximum possible score for each perception and practice domain and multiplying by 100. The higher the score for each domain, the higher the perception and practice towards WPV prevention among healthcare employers. 

Simple linear and multiple linear regression were used to determine factors associated with perception and practice towards WPV prevention. Independent variables included (a) sociodemographic factors such as age, gender, ethnicity, marital status, number of children, and educational level; (b) individual factors such as work experience, WPV prevention training, and communication technique; and (c) organisational factors such as funding, policy, safety procedure, and standard operating procedure (SOP) for reporting WPV. Dependent variables were (a) perception towards WPV prevention and (b) practice towards WPV prevention. Variables with a *p*-value < 0.25 from simple linear regression or important factors from other studies were selected into multiple linear regression analysis. Multiple linear regression was used to examine the impact of multiple risk factors on the outcome. The stepwise process was used to fit regression models for variable selection, which were backward and forward selection procedures. The preliminary main effect model was created at this point. Any interactions between the variables in the preliminary main effect model were investigated. All potential two-way interactions were examined. A correlation matrix and standard error were used to verify multicollinearity. The preliminary final model’s results were then acquired.

The coefficient of multiple determination (R^2^) was used to assess the model’s fitness. The value for R^2^ is 0 ≤ R^2^ ≤ 1. The final model was kept with the biggest numbers of significant predictors, rules of parsimony (the simplest plausible model with the fewest possible number of variables), statistically sound, and biologically plausible. Finally, tables with a crude regression coefficient, adjusted regression coefficient, 95% confidence intervals (CI), t-statistic, and *p*-values were created to display the results. The significance threshold was chosen at 0.05.

### 2.5. Ethical Considerations

This study was undertaken in accordance with the guidelines of the national/international/institutional Declaration of Helsinki. Approval from the Medical Research and Ethics Committee (MREC) of the Ministry of Health Malaysia (NMRR-21-338-57929) and the Human Research Ethics Committee of Universiti Sains Malaysia (USM/JEPeM/21020165) was obtained. A written information and sheet consent form was provided and obtained from all participants. Data confidentiality was firmly preserved and only restricted to the research team.

## 3. Results

### 3.1. Characteristics of Participants

Employers from the hospital were the highest proportion (39.5%), and the most representative of healthcare employers was the location supervisor (71.6%). Most of them are female (63%), Malay (92%), married (88.9%), had less than five children (79%), were diploma holders (49.3%), and had working experience of more than ten years (90.1%) (Table 1).

### 3.2. Perception and Practice towards WPV Prevention among Employers at Healthcare Facilities in Melaka

The summative mean percentage for perception towards WPV prevention was 67.2% (Table 2), whereas for that of practice was 80% (Table 3). The mean percentage for perception was lower than that of practice towards WPV prevention due to low participants’ scoring for eight items in the domain form of WPV. As shown in Table 4, of scoring one (strongly disagree) to five (strongly agree), the mean for item 1 was 2.91 (SD 1.29); item 2 was 2.28 (SD 1.12), item 3 was 2.23 (SD 1.08), item 4 was 2.30 (SD 1.13), item 5 was 2.20 (SD 1.06), item 6 was 2.44 (SD 1.14), item 7 was 1.88 (SD 0.89), and item 8 was 1.99 (SD 0.97).

The mean percentage for practice toward WPV prevention was higher due to high participants’ scoring for most of the items, with a mean score ranging from 2.20 (0.99) to 4.44 (0.65) (Table 5). Among the 4 domains, WPV reporting had a low mean percentage of 46.5% (Table 3). Meanwhile, the other 3 domains: workplace safety, WPV prevention implementation, and managerial role, had a high mean percentage at 82.3%, 82%, and 87.6%, respectively (Table 3). 

### 3.3. Factors Associated with Perception towards WPV Prevention among Employers at Healthcare Facilities in Melaka

#### 3.3.1. Simple Linear Regression Analysis

In the univariable analysis in assessing factors associated with perception towards WPV prevention, there were six variables with a *p*-value of <0.25. The variables were age, gender, ethnicity, number of children, educational level, and enough funding for WPV prevention. The multivariable analysis included those variables to encounter possible confounders (Table 6).

#### 3.3.2. Multiple Linear Regression Analysis

The variable of the number of children was removed and the preliminary main effect model with five remaining variables was obtained in the multivariable analysis after comparing the model using backward and forward linear regression. The five variables were age, gender, ethnicity, educational level, and enough funding for WPV prevention. The model ended up with four significant variables: gender, ethnicity, educational level, and enough funding (Table 6).

All the possible two-way interactions were checked between important independent variables in the main effects model and were found to be not significant. The correlation matrix showed that the dependence between multiple variables at the same time was weakly correlated, indicating no multicollinearity problem exists. The standard error of variables was relatively small, which signifies no multicollinearity. Thus, a preliminary final model was obtained. The fitness of the model was tested by R^2^. The R^2^ value was 0.16, suggesting that the model reasonably fits well. Thus, the model assumptions were met. 

#### 3.3.3. Interpretation of the Final Model

Multiple linear regression analysis showed gender, ethnicity, educational level, and enough funding for WPV prevention have a significant linear relationship to perception towards WPV prevention. The model explains 16% of the variation of perception scores in the study sample. Compared to male, female employers had a lower perception score of 3.95 (95% CI: −7.81, −0.09) when adjusted to other variables. Compared to Malay, Indian employers had a higher perception score towards WPV prevention by 16.04 (95% CI: 2.34, 29.74), while other ethnic had a higher perception score by 25.71 (95% CI: 8.94, 42.47) when adjusted to other variables. Meanwhile, compared to diploma holders, employers with degrees had higher perception scores towards WPV prevention by 4.35 (95% CI: 0.15, 8.54), and employers with a master’s had higher perception scores by 7.63 (95% CI: 1.11, 14.14) when adjusted to other variables. Finally, employers with enough funding for WPV prevention had lower perception scores towards WPV prevention by −5.46 (95% CI: −9.25, −1.67) than those with insufficient funding when adjusted to other variables.

### 3.4. Factors Associated with Practice towards WPV Prevention among Employers at Healthcare Facilities in Melaka

#### 3.4.1. Simple Linear Regression Analysis

In the univariable analysis to assess factors associated with practice towards WPV prevention, there were nine variables with a *p*-value of <0.25. The variables were ethnicity, marital status, number of children, educational level, work experience, enough funding, policy for WPV prevention, safety procedure for WPV, and SOP for reporting WPV episodes. Those variables were included in the multivariable level analysis to encounter possible confounders (Table 7).

#### 3.4.2. Multiple Linear Regression Analysis

The number of children, work experience, enough funding, policy for WPV prevention, and safety procedure for WPV was removed and the preliminary main effect model with the remaining four variables was obtained at the multivariable level after comparing the model using backward and forward linear regression. The four variables were ethnicity, marital status, educational level, and SOP for reporting WPV. Finally, the model ended up with three significant variables: ethnicity, educational level, and SOP for reporting WPV (Table 7).

#### 3.4.3. Interpretation of the Final Model

Multiple linear regression analysis showed ethnicity, educational level, and availability of SOP for reporting WPV have a significant linear relationship to practising WPV prevention. The model explains 12% of the variation of practice scores in the study sample. 

Compared to Malay employers, Chinese employers had lower practice towards WPV prevention by 9.25 (CI: −18.36, −0.14), Indians had lower practice towards WPV prevention by 14.97 (CI: −29.48, −0.46), while other ethnic groups had higher practice towards WPV prevention by 23.55 (CI: 5.59, 41.51), when adjusted to other variables. Compared to diploma holders, employers with degrees had lower practice towards WPV prevention by 4.41 (CI: −8.67, −0.14) when adjusted to other variables. Furthermore, employers with the availability of SOP for reporting WPV at the workplace had higher practice scores towards WPV prevention by 6.07 (CI: 1.58, 10.57) when adjusted to other variables.

## 4. Discussion

It is unfortunate that the prevalence of WPV against HCWs continues to rise due to inconsistent implementation of its prevention. Employers’ attribution is very important in WPV prevention. A cross-sectional study was conducted among employers at healthcare facilities in Melaka using the PPWVP questionnaire. This study aims to determine the level of perception and practice towards WPV prevention, and its associated factors among employers at healthcare facilities in Melaka. It is important because, despite the high prevalence of WPV, there is a negative perception and poor practice of healthcare employers towards WPV prevention. A thorough assessment and discussion on WPV prevention and its associated factors in this section, may provide further evidence for employers to take preventive actions to eliminate WPV, especially in terms of perception and practice towards WPV prevention. This study may help to improve the health of HCWs, enhance patient care provider services, and improve patients’ quality of life.

### 4.1. Perception and Practice towards WPV Prevention among Employers at Healthcare Facilities in Melaka

The employers at healthcare facilities had a high mean percentage of perception towards WPV prevention regarding impacts of WPV, causes of WPV, reaction to WPV, high-strain job characteristics, WPV protection, benefits of WPV prevention, barriers to WPV prevention, and WPV prevention encouragement. Meanwhile, the employers had a low mean percentage of perception towards the form of WPV. In additon, the employers also had a high mean percentage on practice towards WPV prevention regarding workplace safety, WPV prevention implementation, and the managerial role in practicing WPV prevention, whereas there was a low mean percentage on WPV prevention support. Overall, the summative mean percentage for both perception and practice towards WPV prevention was high at 67.2% and 80%, respectively. This high mean percentage may be attributed to the fact that participants mostly had received a good education with at least a diploma holder, sufficient work experience, and were well versed in their current workplace. However, not all participants shared the same perception and practice score towards WPV prevention despite their educational background, indicating the need for improvement in the current WPV interventions.

The participants scored a low mean percentage in the form of WPV. Most participants either disagree or strongly disagree with the existence of violence at their workplace. It can be best explained that WPV episodes used to happen in healthcare facilities, misleading employers to have a low perception towards any form of WPV. In fact, according to the Ministry of Health, 70% of HCWs in Malaysia experienced verbal abuse, 33% physical assault, 25% bullying, and 4% sexual harassment [11]. In a different view, the low perception towards the form of WPV indicates that the prevalence of WPV at healthcare facilities in Melaka is relatively low and below the national prevalence [9,11].

The current study found a few causes of WPV that healthcare employers commonly perceived. This included the expression of the perpetrators’ feelings. This finding is supported by recent evidence, which suggests that personal characteristics and poor anger management of perpetrators are the causes of WPV. Patients and their relatives were aggressive, quick-tempered, and intolerant, causing increased tension between them and HCWs and leading to violence [24]. Most of the participants in the current research agreed on the high-strain job characteristics such as shortage of staff and increased number of patients in healthcare facilities. Previous studies have shown that high numbers of patients at healthcare facilities have caused them not to receive proper medical care and become one of the factors for WPV [24,25].

The majority of participants agreed on the impacts of WPV that injure staff and damage property (93.2%). The finding is congruent with a previous study, which reported that the perceived impact of verbal abuse on HCWs was decreased job satisfaction [26]. In addition, a study by Escribano et al. [27] has highlighted critical issues regarding WPV in the health sector, including the perceived impact of work on health. The study conducted a data review of the European Working Conditions Surveys (EWCS) and demonstrated the negative impact of work on workers’ health and the rate of workers that perceived the negative impact varied over time: 31.1% in all of the European Union in the I EWCS in 1992; 57% in the II EWCS in 1997; 60% in the III EWCS in 2001, and 25% in the IV EWCS in 2016 [27]. Several other studies further support the current findings. They reported that WPV has a significant direct impact [28,29] and an indirect impact [30] on the health sectors. 

Furthermore, most participants also agreed about the reaction to WPV. Many of them agreed that violence in the workplace is a normal reaction to perpetrators’ feelings of anger. In contrast, the existing studies showed different findings. For example, a few studies reported HCWs felt disturbed but knew they had to cope [31] and claimed disbelief and shock [32]. Nevertheless, this again highlighted the importance of acknowledging that each individual reacts differently and is entirely normal [33].

Most participants had a similar view on WPV protection. They agreed on the absence of effective WPV prevention programs and regulations to protect staff. The previous study has shown that managers viewed the influence of organisational policies, rules, and regulations as detrimental to the safety of staff working in healthcare settings [34]. Similarly, Ori et al. [35] highlighted that putting written policies on the walls and other visible areas of healthcare facilities about punishments could help to deter people from committing violence. 

The two most popular benefits of WPV prevention were increased staff awareness about the risk of violent incidents at the workplace and improved staff safety. These findings are congruent with Hanson et al. [36], which reported that the initiative to address WPV was able to reduce burnout, develop a safer workplace, and enable the optimum provision of health services. In contrast, participants had different views on the barriers to WPV prevention in the current study. A high percentage of participants show neutral opinions or disagreements about financial, time, and human resource constraints, which are considered barriers to WPV prevention. The results are incongruent with the previous study, which considered the above constraints as barriers to WPV prevention [37,38]. Current research only focused on the government employers that are already equipped with enough resources and support from the government. 

Regarding WPV prevention encouragement, many participants were uncertain whether the increased cost of treatment and compensation and loss of work time could encourage WPV prevention programs. A study on WPV in healthcare environments in the United States highlighted that hospitals are the most likely places for violent episodes that result in time lost from work [39]. The time lost from work among HCWs means a time loss for patient care and could compromise standard medical care [30,40]. WPV in healthcare facilities has direct financial impacts due to subsequent litigation from the involved parties, whereas indirect financial impacts for facilities see a higher turnover rate and absconding from duty [41]. Both time loss for patient care and increased financial impacts due to WPV has led to its prevention initiative.

Most participants agreed (mean percentage: 82.3%) upon workplace safety practices, including electronic observation, security guards, and physical safety protection. The finding is promising and consistent with previous studies. A study conducted in a Colorado detoxification facility in the United States to investigate the results of the WPV prevention program found that increased visibility of electronic surveillance, such as closed-circuit television, can reduce WPV [42]. Meanwhile, Lipscomb et al. [43] have reported that physical violence with injury could be reduced by replacing solid panel doors with transparent panels.

Current findings show that participants scored a high mean percentage (82%) in WPV prevention implementation. One of the most popular practices was ensuring that workplace organisations have policies that include the prevention of WPV. This is in line with Ferris and Murphy [19], which suggested formal policy as part of a comprehensive WPV prevention strategy. Organisations with formal WPV policies were found to have a higher awareness of WPV issues than organisations without policies. Unfortunately, while WPV policies are zero tolerance in principle, employers may fail to enforce them [44]. The inherence of a formal WPV policy may signal that organisations are more concerned about protecting their workers and customers from WPV events [44]. The argument highlighted that the policy for WPV prevention demands that the employers and organisations give support to their workers at the workplace.

The mean percentage for WPV reporting was low at 46.5%. Most participants disagreed with the two items, ‘Over the past 12 months, incidents of workplace violence at my organisation have increased’ and ‘Over the past 12 months, incidents of workplace violence have affected staff in my organisation’. This finding showed that activities to report WPV cases were carried out, but the cases could still be underreported. This is comparable to a study by Alahmadi & Makhdoom [45], which demonstrated that less than 32% of HCWs reported violent events to their supervisors. Reporting is important in preventing future WPV cases, and healthcare employers should fully support it. Furthermore, Arnetz et al. [46] have highlighted that incident reporting would help identify the causes of WPV and suggest a necessary improvement. 

A study was conducted among HCWs in all job positions to examine the episodes of WPV experienced by HCWs working in Italian public hospitals. It was found that male HCWs aged less than 30 did not report WPV events, and male HCWs reported more violent episodes than their female counterparts [47]. Other reasons for underreported WPV cases include HCWs being more likely to report serious events and exclude less serious ones [48], employers having difficulty using formal reporting systems [22], and workplaces did not have procedures for reporting violence [49]. The arguments demonstrated that the WPV reporting measure has not yet been consistently implemented.

The mean percentage for the managerial role in practising WPV prevention was 87.6%. Most of the participants agreed and strongly agreed with the listed managerial role, such as taking appropriate action when bullying occurs at work (91.3%), creating a safe workplace environment (93.9%), and accepting staff opinions about WPV prevention programs or policies (92.6%). Current findings are comparable to the previous study, which showed that employers regularly evaluate employees’ work performances, and allowing opportunities to discuss grievances was crucial to develop WPV prevention [21]. However, another study reported an incongruent finding in which the employers did not encourage staff to do formal incident reporting post-violence at the workplace [22]. Employers see increased reports made by employees as an extra workload [50]. These differences show employers’ crucial role in positive or negative ways in WPV prevention.

### 4.2. Factors Associated with Perception towards WPV Prevention among Employers at Healthcare Facilities in Melaka

In the current study, four factors were identified to be significantly associated with perception towards WPV prevention. The factors were female, Indian and other ethnicities, educational level, and enough funding for WPV prevention. 

First, the gender of healthcare employers showed statistical significance for perception towards WPV prevention. Female employers had a lower perception score towards WPV prevention by 3.95 (95% CI: −7.81, −0.09) compared to males when adjusted to other variables. WPV prevention is a new recommended policy at the workplace, and it is understandable if employers have difficulty in making a decision to go for or against this new policy. It is possible that female employers are perceived as less likely to execute new policies such as WPV prevention, given the numerous barriers mentioned earlier in the current study. This is comparable to a study done among youth, adults, and retired people from various professional’s backgrounds in Spain, which reported significant gender differences in decision-making because females were more concerned about uncertainty, doubts, and dynamism involved in the decision. Moreover, females put greater value on money and time and were more worried about the implication of decisions, regardless of whether these affected them or others [51]. This contrasts with a study done among professional managers in Finland to examine workplace bullying. The study has reported that a female is more likely than a male to regard workplace bullying as an organisational issue [52]. Enhanced recognition of WPV issues by employers is critical for its prevention [53]. Another study conducted among supervisors in the United States to investigate supervisors’ perceptions of intimate partner violence among a diverse workforce. The study has highlighted that intervention by supervisors is required each time employees are harassed or abused by their intimate partners while at work. However, male supervisors expressed concern about being accused of sexual harassment if they intervened to support the employees, resulting in a poor perception towards WPV prevention [54]. 

Second, the ethnicity of healthcare employers showed statistical significance for perception towards WPV prevention. Compared to Malay employers, Indians had a higher perception score of 16.04 (95% CI: 2.34, 29.74), while other ethnic groups had a higher perception score of 25.71 (95% CI: 8.94, 42.47) when adjusted to other variables. This study found that Indian and other ethnic groups have a higher perception of WPV prevention than Malay employers. However, few past studies reported contradictory findings that ethnicity has no significant influence on WPV [55,56]. Regardless, previous studies have reported that justice, law enforcement, and healthcare service responses to violence are unfairly influenced by ethnic group [57,58].

Malaysia is a multiracial country where different ethnicities mix and interact harmonically. However, each ethnic group has diverse cultures that could manifest differently in the workplace, such as their language, culture, religion, and adaptation to organisational needs. Indian and other ethnic groups of employers have been shown to have better perceptions towards WPV prevention, suggesting they adapted to organisational culture more successfully than Malays. Furthermore, a previous study has shown that when the organisational culture and values are found unsuitable for workers, the employers should try to understand and appreciate the differences [59]. Moreover, the majority of the participants in this study were Malays. Abdullah et al. [60] have highlighted that the Malay cultural ideals of harmony between employers and employees include cooperation, tolerance, avoiding conflicts, and mutual respect, thus, emphasising a crucial need for successfully implementing the WPV prevention program. Ultimately, successful WPV prevention implemented at the workplace is proven to increase the company’s competitiveness [60].

Third, the current study found that healthcare employers with a degree and master’s education have a higher perception of WPV prevention than diploma holders, indicating that education plays a role in preventing WPV. Compared to diploma holders, employers with degrees had higher perception scores by 4.35 (95% CI: 0.15, 8.54), and employers with master’s had higher perception scores by 7.63 (95% CI: 1.11, 14.14) when adjusted to other variables. This is comparable to a previous study, which reported that the prevalence of WPV varies by educational level. For example, nurses with graduate degrees report a higher prevalence (84.6%) compared to 76.7% of nurses with undergraduate degrees. The findings suggest that nurses with graduate degrees have more heightened rights-protection awareness, do not regard assaults as ‘part of the job’, and have no fear that reporting violence will negatively impact their external evaluation [61]. 

In addition, the current study found that employers with sufficient financial resources have reduced perceptions towards WPV prevention. Healthcare employers with enough funding for WPV prevention had lower perception scores by 5.46 (95% CI: −9.25, −1.67) than those with insufficient funding when adjusted to other variables. One possible reason could be that the participants considered the WPV prevention policy to be already incorporated into their workplace policy. This argument is supported by the results of the practice domain, in which 83% of participants agreed that ‘I ensure that workplace organisations have programs or policies that include the prevention of WPV’. Another explanation is that all the participants were government servants. Therefore, in implementing any policy or program, the financial constraints are irrelevant because the organisation must use and adapt to the existing funds, be it enough or not. 

Moreover, most employers could have perceived that WPV prevention execution is not expensive or require too much cost. The current finding is comparable to the conclusions by Ferris and Murphy [19] and Morphet et al. [20], in which they did not recommend sufficient financial support to be one of the essential components of the WPV prevention strategy. Nevertheless, Awadalla and Roughton [62] demonstrated different findings where they reported that successful WPV prevention does require enough financial support.

### 4.3. Factors Associated with Practice towards WPV Prevention among Employers at Healthcare Facilities in Melaka

The final model following multivariable analyses shows three variables significantly associated with practice towards WPV prevention: Chinese, Indian, and other ethnicities; degree holders; and SOP for reporting WPV.

First, the ethnicity of healthcare employers showed statistical significance for practice towards WPV prevention. Compared to Malay employers, the Chinese had a lower practice score towards WPV prevention by 9.25 (CI: −18.36, −0.14), Indians had a lower practice score towards WPV prevention by 14.97 (CI: −29.48, −0.46), while other ethnic groups had higher practice score towards WPV prevention by 23.55 (CI: 5.59, 41.51) when adjusted to other variables. The findings suggest that healthcare employers with an ethnicity other than Malay have different ways of adapting to organisational culture. There has been less study of whether and how ethnicity differentially impacts WPV prevention, but this difference in ethnicity can be compared to other studies. For example, Chinese and Indian employers tend to have poor adaptation, whereas other ethnic employers show better adaptation to organisational culture. Asma Abdullah [59] supports this argument based on her report stating that when organisational values are unsuitable for workers due to their different values and culture, employers should take time to understand workers and appreciate the differences. 

It is essential to emphasize that Chinese, Indian, and other ethnic groups were the minority in the current study. A study was conducted among workers from various ethnic groups: Hispanic, Anglo, and African-American, in the United States to examine their differences in response patterns on workplace questionnaires. It was found that Hispanics, being the minority group [63], tended to report fewer negative job behaviours such as job stress, antagonistic work behaviours, and psychological withdrawal [64]. Another study highlighted that increased resource utilisation and lower risk of WPV among minority workers were related to services addressing previous trauma, education, and training opportunities for the fresh employee [65].

Second, the current study showed that being a degree holder is statistically significant for WPV prevention practice. Compared to diploma holders, healthcare employers with degrees had lower practice scores towards WPV prevention by 4.41 (95% CI: −8.67, −0.14) when adjusted to other variables. Meanwhile, healthcare employers with master’s and PhD levels of education are found statistically insignificant to the practice towards WPV prevention. The explanation would be that most participants have acknowledged the availability of the WPV prevention policy at their workplace, and almost half of the participants in this study are diploma holders (49.3%). Thus, it can be postulated that healthcare employers are committed to practicing WPV prevention regardless of educational background. Theoretically, the higher the education, the higher the practice towards WPV prevention. This is proved by a study in Chinese hospitals, which reported the relationship between higher educational level and reporting for WPV events. Reporting is a critical component in preventing future WPV, and the study showed that WPV prevalence increases among nurses with graduate degrees (84.6%) compared to nurses with undergraduate degrees 76.7% [61]. The study explained that nurses with graduate degrees neither consider assaults as a norm nor part of the job, have higher rights-protection awareness, and have no fear that reporting violence will negatively impact their external evaluation [61].

Third, the current study showed that having SOP for reporting WPV is a significant predictor for better practice towards WPV prevention. The availability of SOP for reporting WPV incidents at the workplace had a higher practice score towards WPV prevention by 6.07 (95% CI: 1.58, 10.57) when adjusted to other variables. The finding proved that reporting violent incidents is essential to overcome WPV because it permits the prevention program to identify violence issues within healthcare facilities. However, several pieces of literature have reported issues which include the unavailability of proper measures for incident reporting, and reporting is pointless as it does not result in effective corrective actions, leading to underreporting of WPV cases [22,38,66]. It is crucial for WPV prevention programs to acknowledge and act accordingly on every report to address the victims’ concerns and show that management is interested in correcting the reported concern. Therefore, HCWs would proceed with an official report for every WPV incident if there is a proper reporting system because the availability of the SOP would help them quickly follow the steps. This argument is in agreement with Li et al. [49], who emphasised that HCWs knew how to use reporting system when it was readily available at their workplace.

### 4.4. Strengths and Limitations

The major strength of this study is the inclusion of three different levels of employers, including the director of the organisation, location supervisor, and OSHC. Therefore, the research outcome concerning the preventive measures of WPV prevention could be introduced at various or multilevels, including the whole organisation, departmental, or unit levels, in order to recognise vulnerable employees or perpetrators with the risk of exposure to violence. Furthermore, this study involved five categories of healthcare facilities: hospitals, health clinics, dental clinics, district health offices, and district dental offices. Despite the majority of the participants being from hospitals, the heterologous study site used in this study ensures data generalizability and representativeness, which counted as another research strength. In addition, the determination of perception and practice towards WPV prevention in this study used a locally validated Malay language questionnaire. Thus, the findings truly represent the local population, the healthcare employers in Melaka, Malaysia.

The main limitation encountered in this research is the stigma of being blamed and Malaysian healthcare’s punitive culture. This could discourage participants from answering sensitive questions because they fear the blaming culture, although confidentiality and anonymity are explicitly mentioned in this study [67,68]. Another limitation was the surge of COVID-19 cases during the pandemic. The ever-busy healthcare employers, the hectic situation in healthcare facilities, and COVID-19 management time constraints could invite bias among the participants while filling out the questionnaire.

## 5. Conclusions

The current study explored the perception and practice of WPV prevention among employers at healthcare facilities using the locally validated PPWVP questionnaire. The healthcare employers had relatively high scores (mean percentage 67.2%) for perception towards WPV prevention with regards to the form of WPV, impacts of WPV, causes of WPV, reaction to WPV, high-strain job characteristics, WPV protection, benefits of WPV prevention, barriers to WPV prevention, and WPV prevention encouragement. In addition, the employers had a relatively high score (mean percentage 80%) for practice towards WPV prevention regarding workplace safety, WPV prevention implementation, WPV reporting, and the managerial role in practising WPV prevention. 

Several factors were found to be associated with healthcare employers’ perception and practice toward WPV prevention. For example, female, Indian and other ethnicities, degree and master’s holders, and enough funding for WPW prevention are the significant factors for the perception of WPV prevention among healthcare employers. Meanwhile, Chinese, Indian and other ethnicities, degree holders, and the availability of standard operating procedures for reporting WPV are significant factors for healthcare employers’ practice towards WPV prevention. Hence, this study recommends the inclusion of these factors to improve the existing guideline and training module of the Ministry of Health Malaysia for WPV prevention in healthcare facilities.

## Figures and Tables

**Table 1:** Characteristics of employers at healthcare facilities in Melaka in a cross-sectional study (*n* = 162).

Variables		*n* (%)	Mean (SD)
Age (years)			43.90 (7.80)
Gender			
	Male	60 (37)	
	Female	102 (63)	
Ethnicity			
	Malay	149 (92.0)	
	Chinese	8 (4.9)	
	India	3 (1.9)	
	Others	2 (1.2)	
Marital status			
	Single	9 (5.5)	
	Married	144 (88.9)	
	Divorced	9 (5.5)	
Number of children			
	No child	23 (14.2)	
	Less than five	128 (79.0)	
	Five and more	11 (6.8)	
Educational level			
	Diploma	80 (49.3)	
	Degree	63 (38.9)	
	Master’s	16 (9.9)	
	PhD	3 (1.9)	
Work experience			
	Less than 10 years	26 (16.0)	
	More than 10 years	146 (90.1)	
Ever had WPV prevention training (Yes)		32 (19.8)	
Communication technique (Good)		320 (96.1)	
Types of workplaces			
	Hospitals	64 (39.5)	
	Health clinics	47 (29.0)	
	Dental clinics	25 (15.4)	
	District health offices	24 (14.8)	
	District dental offices	2 (1.2)	
Level of Employer			
	Director of the organisation	22 (13.6)	
	Location supervisor	116 (71.6)	
	OSHC *	24 (14.8)	
Organisational factors for WPV prevention			
	Enough funding (Yes)	75 (46.3)	
	Policies (Yes)	108 (66.7)	
	Safety procedure (Yes)	101 (62.3)	
	SOP for reporting ^¥^ (Yes)	117 (72.2)	

* OSHC = Occupational Safety & Health Committee. ^¥^ SOP = Standard operating procedure.

**Table 2:** Mean percentage of perception towards WPV prevention domain in PPWVP (*n* = 162).

No	Domain	Perception Score			
		Min	Max	Mean (SD)	Mean %
1	Form of workplace violence	8	40	18.24 (6.81)	45.6%
2	Benefits of workplace violence prevention	5	25	21.96 (3.34)	87.8%
3	Barriers to workplace violence prevention	5	25	15.25 (3.94)	61%
4	Impacts of workplace violence	2	10	9.45 (1.43)	94.5%
5	Causes of workplace violence	3	15	10.40 (1.97)	69.3%
6	Reaction to workplace violence	3	15	11.19 (3.00)	74.6%
7	High-strain job characteristics	3	15	10.19 (3.31)	67.9%
8	Workplace violence protection	2	10	7.07 (2.13)	70.7%
9	Workplace violence prevention encouragement	2	10	7.21 (1.66)	72.1%
	Summative score	33	165	110.96 (12.77)	67.2%

**Table 3:** Mean percentage of practice towards WPV prevention domain in PPWVP (*n* = 162).

No	Domain	Practice Score			
		Min	Max	Mean (SD)	Mean %
1	Workplace safety	3	15	12.35 (2.47)	82.3%
2	Workplace violence prevention implementation	15	75	61.84 (10.22)	82%
3	Workplace violence reporting	2	10	4.65 (1.97)	46.5%
4	Managerial role	3	15	13.14 (1.67)	87.6%
	Summative score	23	115	91.97 (13.3)	80%

**Table 4:** Perception towards WPV prevention domain in PPWVP (*n* = 162).

No	Variables	Answer Response, *n* (%)					Mean (SD)
		Strongly Agree	Agree	Neutral	Disagree	Strongly Disagree	
Form of workplace violence							18.24 (6.81)
1	I believe there has been verbal intimidation in the workplace over the past year	19 (11.7)	41 (25.3)	39 (24.1)	33 (20.4)	30 (18.5)	2.91 (1.29)
2	I believe there has been physical violence in the workplace over the past year	10 (6.2)	10 (6.2)	40 (24.7)	57 (35.2)	45 (27.8)	2.28 (1.12)
3	I believe there has been an act of vandalism in my workplace over the past year	4 (2.5)	19 (11.7)	37 (22.8)	53 (32.7)	49 (30.2)	2.23 (1.08)
4	I believe there have been attempted physical assaults on my staff over the past year	8 (4.9)	16 (9.9)	38 (23.5)	55 (34.0)	45 (27.8)	2.30 1.13)
5	I believe there has been sexual harassment at the workplace over the past year	4 (2.5)	13 (8.0)	48 (26.6)	44 (27.2)	53 (32.7)	2.20 (1.06)
6	I believe there have been acts of bullying in the workplace over the past year	4 (2.5)	32 (19.8)	36 (22.2)	49 (30.2)	41 (25.3)	2.44 (1.14)
7	I believe there has been racial harassment (racist) in the workplace over the past year	1 (0.6)	9 (5.6)	22 (13.6)	68 (42.0)	62 (38.3)	1.88 (0.89)
8	I believe there have been acts of stalking staff at work over the past year	2 (1.2)	9 (5.6)	35 (21.6)	55 (34.0)	61 (37.7)	1.99 (0.97)
Benefits of workplace violence prevention							21.96 (3.34)
9	Prevention of workplace violence improves the safety of staff	96 (59.3)	55 (34.0)	8 (4.9)	1 (0.6)	2 (1.2)	4.49 (0.73)
10	Prevention of workplace violence can increase staff awareness of the risk of violent incidents in the workplace	91 (56.2)	0	63 (38.9)	7 (4.3)	1 (0.6)	4.50 (0.64)
11	Prevention of workplace violence can reduce the cost of treatment that has to be borne as a result of the violent cases that occur	74 (45.7)	63 (38.9)	17 (10.5)	6 (3.7)	2 (1.2)	4.24 (0.88)
12	Prevention of violence in the workplace can reduce the cost of compensation to be incurred as a result of the violent cases that occur	77 (47.5)	66 (40.7)	15 (9.3)	3 (1.9)	1 (0.6)	4.33 (0.77)
13	Prevention of workplace violence will improve the image of the organisation	87 (53.7)	62 (38.3)	7 (4.3)	3 (1.9)	3 (1.9)	4.40 (0.82)
Barriers to workplace violence prevention							15.25 (3.94)
14	I have limited time to implement workplace violence prevention programs	6 (3.7)	49 (30.2)	43 (26.5)	49 (30.2)	15 (9.3)	3.11 (1.06)
15	I have financial constraints in implementing workplace violence prevention programs	5 (3.1)	23 (14.2)	62 (38.3)	48 (29.6)	24 (14.8)	3.39 (1.01)
16	I have staff constraints to implementing workplace violence prevention programs	7 (4.3)	37 (22.8)	37 (22.8)	56 (34.6)	25 (15.4)	3.34 (1.12)
17	Staff working in remote areas is an obstacle to workplace violence prevention programs	16 (9.9)	62 (38.3)	49 (30.2)	27 (16.7)	8 (4.9)	2.69 (1.02)
18	Staff working shifts are an obstacle to workplace violence prevention programs	15 (9.3)	67 (41.4)	40 (24.7)	28 (17.3)	12 (7.4)	2.72 (1.10)
Impacts of workplace violence							9.45 (1.43)
19	Violence in the workplace is an act that should not be accepted	139 (85.8)	15 (9.3)	3 (1.9)	1 (0.6)	4 (2.5)	4.75 (0.75)
20	I accept violence at the workplace can injure staff and damage property in the workplace	132 (81.5)	19 (11.7)	6 (3.7)	2 (1.2)	3 (1.9)	4.70 (0.77)
Causes of workplace violence							10.40 (1.97)
21	Violence in the workplace is an expression of a patient’s or visitor’s feelings, much like anger or growling	23 (14.2)	50 (30.9)	27 (16.7)	22 (13.6)	40 (24.7)	2.96 (1.42)
22	After committing violence in the workplace, patients or visitors feel calmer *	62 (38.3)	38 (23.5)	45 (27.8)	13 (8.0)	4 (2.5)	3.87 (1.10)
23	Violence in the workplace is one of the methods of the perpetrator to protect himself *	48 (29.6)	37 (22.8)	46 (28.4)	21 (13.0)	10 (6.2)	3.57 (1.22)
Reaction to workplace violence							11.19 (3.00)
24	Violence at the workplace is a normal reaction to feelings of anger *	41 (25.3)	37 (22.8)	25 (15.4)	41 (25.3)	18 (11.1)	3.26 (1.37)
25	Violence in the workplace is a positive reaction caused by the anger of the patient or visitors while receiving treatment/running errands *	69 (42.6)	36 (22.2)	26 (16.0)	18 (11.1)	13 (8.0)	3.80 (1.31)
26	Workplace violence can help staff to improve the relationship between staff and patients *	88 (54.3)	35 (21.6)	19 (11.7)	11 (6.8)	9 (5.6)	4.12 (1.19)
High-strain job characteristics							10.19 (3.31)
27	Workplace violence is caused by a shortage of staff working at the scene	25 (15.4)	51 (31.5)	34 (21.0)	39 (24.1)	13 (8.0)	3.22 (1.21)
28	Workplace violence occurs due to an increase in the number of patients or the occurrence of overcrowding at work	40 (24.7)	60 (37.0)	27 (16.7)	25 (15.4)	10 (6.2)	3.59 (1.19)
29	Workplace violence prevails due to staff working in small numbers (less than 5 people)	33 (20.4)	50 (30.9)	36 (22.2)	31 (19.1)	12 (7.4)	3.38 (1.22)
Workplace violence protection							7.07 (2.13)
30	Workplace violence occurs due to the absence of an effective workplace violence prevention program	31 (19.1)	59 (36.4)	37 (22.8)	22 (13.6)	13 (8.0)	3.45 (1.18)
31	Workplace violence occurs due to the absence of regulation to protect staff	46 (28.4)	57 (35.2)	22 (13.6)	25 (15.4)	12 (7.4)	3.62 (1.25)
Workplace violence prevention encouragement							7.21 (1.66)
32	Increased costs of treatment and workers’ compensation may drive the implementation of workplace violence prevention	32 (19.8)	62 (38.3)	51 (31.5)	13 (8.0)	4 (2.5)	3.65 (0.97)
33	The time lost to patient care and work can encourage the implementation of workplace violence prevention	25 (15.4)	62 (38.3)	57 (35.2)	16 (9.9)	2 (1.2)	3.57 (0.91)

* Statement = reverse statement.

**Table 5:** Practice towards WPV prevention domain in PPWVP (*n* = 162).

No	Variables	Answer Response, *n* (%)					Mean (SD)
		Strongly Agree	Agree	Neutral	Disagree	Strongly Disagree	
Workplace safety							12.35 (2.47)
1	I ensure electronic observation is provided at the workplace	61 (37.7)	64 (39.5)	14 (8.6)	16 (9.9)	7 (4.3)	3.96 (1.12)
2	I ensure security guards are provided at the workplace.	78 (48.1)	63 (38.9)	12 (7.4)	7 (4.3)	2 (1.2)	4.28 (0.87)
3	I ensure that physical safety protection is provided at the workplace	62 (38.3)	64 (39.5)	27 (16.7)	8 (4.9)	1 (0.6)	4.10 (0.89)
Workplace violence prevention implementation							61.84 (10.22)
4	I ensure workplace organisations have the power to arrest or detain individuals to be handed over to the police	58 (35.8)	50 (30.9)	42 (25.9)	9 (5.6)	3 (1.9)	3.93 (1.00)
5	I ensure that workplace organisations have the power to confiscate any weapons brought into the workplace area	63 (38.9)	49 (30.2)	37 (22.8)	10 (6.2)	3 (1.9)	3.98 (1.02)
6	I ensure that workplace organisations have mechanisms or methods to identify patients or visitors who have a record of workplace violence	51 (61.5)	60 (37.0)	37 (22.8)	10 (6.2)	4 (2.5)	3.89 (1.00)
7	I ensure that workplace organisations provide additional safety arrangements (e.g., security alarms, physical barriers at workplace stations) for staff who have been victims of workplace violence	59 (36.4)	61 (37.7)	32 (19.8)	6 (3.7)	4 (2.5)	4.02 (0.97)
8	I ensure that workplace organisations have programs or policies that include the prevention of workplace violence	71 (43.8)	64 (39.5)	23 (14.2)	3 (1.9)	1 (0.6)	4.24 (0.81)
9	I ensure that the issue of patients or visitors who are perpetrators of violence is included in the workplace violence prevention policy	64 (39.5)	72 (44.4)	19 (11.7)	6 (3.7)	1 (0.6)	4.19 (0.83)
10	I ensure that the workplace organisation teaches staff to report incidents of violence in the workplace	84 (51.9)	62 (38.3)	12 (7.4)	3 (1.9)	1 (0.6)	4.39 (0.76)
11	I ensure that workplace organisation deals with violent incidents that occur outside the workplace if they are related to duty and the workplace (e.g., harassment, stalking, physical injury or verbal threats)	70 (43.2)	65 (40.1)	21 (13.0)	3 (1.9)	3 (1.9)	4.21 (0.87)
12	I ensure that the workplace organisation periodically reviews the effectiveness of workplace violence prevention programs or policies	56 (34.6)	71 (43.8)	32 (19.8)	2 (1.2)	1 (0.6)	4.10 (0.80)
13	I ensure that the workplace organisation has a dedicated committee or work team that manages the prevention of violence in the workplace	59 (36.4)	66 (40.7)	32 (19.8)	4 (2.5)	1 (0.6)	4.10 (0.84)
14	I ensure the workplace organisation provides staff with workplace violence prevention information materials	61 (37.7)	75 (46.3)	21 (13.0)	4 (2.5)	1 (0.6)	4.18 (0.79)
15	I ensure the workplace organisation provides workplace violence prevention training to staff	59 (36.4)	67 (41.4)	31 (19.1)	3 (1.9)	2 (1.2)	4.10 (0.86)
16	I ensure the workplace organisation provides separate/additional training on domestic violence prevention if needed	48 (29.6)	66 (40.7)	35 (21.6)	11 (6.8)	2 (1.2)	3.91 (0.94)
17	I ensure that staff in my organisation who have experienced any incidents of workplace violence lodge a report, including those who have not suffered injuries	76 (46.9)	72 (44.4)	12 (7.4)	0	2 (1.2)	4.37 (0.68)
18	I ensure that workplace violence prevention programs or policies in the organisation improve after any incident of workplace violence	63 (38.9)	79 (48.8)	16 (9.9)	3 (1.9)	1 (0.6)	4.23 (0.75)
Workplace violence reporting							4.65 (1.97)
19	Over the past 12 months, incidents of workplace violence at my organisation have increased	6 (3.7)	8 (4.9)	38 (23.5)	71 (43.8)	39 (24.1)	2.20 (0.99)
20	Over the past 12 months, incidents of workplace violence have affected staff in my organisation	7 (4.3)	21 (13.0)	40 (24.7)	63 (38.9)	31 (19.1)	2.44 (1.01)
Managerial role							13.14 (1.67)
21	I take appropriate action when bullying occurs at work	66 (40.7)	82 (50.6)	12 (7.4)	0	2 (1.2)	4.31 (0.66)
22	I created a safe workplace environment	84 (51.9)	68 (42.0)	68 (42.0)	8 (4.9)	2 (1.2)	4.44 (0.65)
23	I accept staff opinions about workplace violence prevention programs or policies	74 (45.7)	76 (46.9)	12 (7.4)	0	0	4.38 (0.62)

**Table 6:** Factors associated with perception towards WPV prevention among employers at healthcare facilities in Melaka (*n* = 162).

Variables		SLR ^a^		MLR ^b^		
		Crude ß ^c^ (95% CI)	*p*-Value	Adjusted ß ^d^ (95% CI) ^e^	t-Stat	*p*-Value
Age (years)		−0.24 (−0.49, 0.02)	0.065	−0.22 (−0.48, 0.03)	−1.726	0.086
Gender (female)		−3.40 (−7.49, 0.68)	0.101	−3.95 (−7.81, −0.09)	−2.024	0.045
Ethnicity						
	Malay	0				
	Chinese	−0.43 (−9.38, 8.52)	0.925	−4.02 (−12.64, 4.61)	−0.920	0.359
	Indian	14.24 (−0.14, 28.62)	0.052	16.04 (2.34, 29.74)	2.313	0.022
	Others	23.07 (5.52, 40.62)	0.010	25.71 (8.94, 42.47)	3.029	0.003
Number of children						
	No child	0				
	Less than five	0.29 (−5.40, 6.00)	0.918			
	Five and more	−6.04 (−15.27, 3.19)	0.198			
Educational level						
	Diploma	0				
	Degree	4.59 (0.42, 8.76)	0.031	4.35 (0.15, 8.54)	2.048	0.042
	Master’s	8.50 (1.72, 15.28)	0.014	7.63 (1.11, 14.14)	2.311	0.022
	PhD	7.81 (−6.75, 22.37)	0.291	6.50 (−7.33, 20.33)	0.929	0.355
Enough funding for WPV prevention (Yes)		−5.66 (−9.54, −1.77)	0.005	−5.46 (−9.25, −1.67)	−2.845	0.005

^a^ simple linear regression; ^b^ multiple linear regression; ^c^ crude regression coefficient; ^d^ adjusted regression coefficient (R^2^ = 0.16); ^e^ CI = confidence interval.

**Table 7:** Factors associated with practice towards WPV prevention among employers at healthcare facilities in Melaka (*n* = 162).

Variables		SLR ^a^		MLR ^b^		
		Crude ß ^c^ (95% CI)	*p*-Value	Adjusted ß ^d^ (95% CI) ^e^	t-Stat	*p*-Value
Ethnicity						
	Malay	0				
	Chinese	−9.43 (−18.68, −0.18)	0.046	−9.25 (−18.36, −0.14)	−2.005	0.047
	Indian	−14.09 (−28.95, 0.76)	0.063	−14.97 (−29.48, −0.46)	−2.038	0.043
	Others	21.57 (3.44, 39.71)	0.020	23.55 (5.59, 41.51)	2.591	0.011
Marital status						
	Married	0				
	Single	−6.35 (−15.33, 2.62)	0.164	−6.57 (−15.19, 2.05)	−1.507	0.134
	Divorced	−6.58 (−15.55, 2.40)	0.150	−6.24 (−14.82, 2.34)	−1.437	0.153
Number of children						
	No child	0				
	Less than five	5.04 (−0.89, 10.97)	0.095			
	Five and more	2.34 (−7.25, 11.96)	0.629			
Educational level						
	Diploma	0				
	Degree	−4.74 (−9.14, −0.34)	0.035	−4.41 (−8.67, −0.14)	−1.680	0.043
	Master’s	0.28 (−6.88, 7.43)	0.940	0.84 (−6.11, 7.78)	0.409	0.812
	PhD	−3.52 (−18.87, 11.84)	0.652	−4.16 (−18.71, 10.39)	−0.381	0.573
Work experience (More than 10 years)		3.99 (−1.61, 9.60)	0.161			
Enough funding		2.44 (−1.69, 6.58)	0.245			
Policies (Yes)		3.84 (−0.51, 8.19)	0.083			
Safety procedure (Yes)		4.26 (0.04, 8.48)	0.048			
SOP for reporting (Yes)		4.76 (0.19, 9.32)	0.041	6.07 (1.58, 10.57)	2.669	0.008

^a^ simple linear regression; ^b^ multiple linear regression; ^c^ crude regression coefficient; ^d^ adjusted regression coefficient (R^2^ = 0.12); ^e^ CI = confidence interval.

## Data Availability

The data are not publicly available due to privacy and confidentiality. However, restrictions apply to the availability of the healthcare facilities’ data and are available from the authors with the permission of the organization.
